# Synthesis and Characterization of Composite Materials Based on Sodium Humate and Poly(vinyl alcohol)

**DOI:** 10.3390/polym17081022

**Published:** 2025-04-10

**Authors:** Alma Khassenovna Zhakina, Yevgeniy Petrovich Vassilets, Oxana Vasilievna Arnt, Almat Maulenuly Zhakin, Zeinulla Muldakhmetovich Muldakhmetov

**Affiliations:** Limited Liability Partnership “Institute of Organic Synthesis and Coal Chemistry of the Republic of Kazakhstan”, 100008 Karaganda, Kazakhstan; vassilets88@mail.ru (Y.P.V.); oxana230590@mail.ru (O.V.A.); zhakin-almat@mail.ru (A.M.Z.); iosu@mail.ru (Z.M.M.)

**Keywords:** macromolecular design, sodium humate, poly(vinyl alcohol), gelation, composite material

## Abstract

This article presents a method for synthesizing a polymer composite based on the interaction of PVA and HNa isolated from coals from the Shubarkol deposit (Karaganda, Kazakhstan). The study focuses on the macromolecular aspects of the formation of the polymer matrix structure and the effect of a natural modifier on the properties of the composite. Taking into account the concept of macromolecular design, the addition of small additives of HNa (2–10%) significantly changes the nature of intermolecular interactions in the solution, promoting the accelerated structuring of the polymer network. This is manifested in a decrease in the gelation time, which is confirmed by a kinetic analysis based on changes in the relative viscosity of the systems. It was found that the greatest increase in viscosity is achieved on the fifth day with a content of 10% HNa and pH = 7, which, on the fifth day, indicates a critical concentration of the modifier necessary for the formation of a stable spatial network of hydrogen bonds and ion-dipole interactions between the functional groups of PVA and HNa. Morphological studies using AFM showed that an increase in the HNa content leads to a significant smoothing of the composite surface, indicating the formation of a more homogeneous and dense structure. These changes are due to the reorganization of the macromolecular architecture under the influence of modifying additives. The adsorption characteristics of the composite were estimated by the maximum sorption capacity, which was 3.40 mmol/g for Cu(II) ions. The results emphasize that the targeted control of the structure at the macromolecular level allows the creation of polymeric materials with specified physicochemical properties that are effective for wastewater treatment from heavy metals. The study demonstrates the potential of macromolecular design as a tool for the development of polymer composites with improved performance characteristics and environmental significance.

## 1. Introduction

Currently, studies on the patterns of self-organization of polymer mixtures leading to the formation of composites are of great interest [1,2,3]. Due to these processes, new composite structures are formed with improved characteristics compared to the original polymers. The basis of self-organization are various types of interactions, such as non-covalent, van der Waals, electrostatic, hydrogen, and hydrophobic bonds of macromolecules. They are used to produce synthetic membranes with highly selective transmittance, ion-exchange composites, and carriers of physiologically active substances and drugs, in particular, sorbents, etc. Polyelectrolyte-based composites are particularly promising, since the physical and mechanical properties of such composites depend not only on the density of the mesh and temperature, but also on the pH and ionic strength of the solution. These circumstances provide additional possibilities for regulating the properties of composites.

One of the simplest methods for obtaining such composites is mixing solutions of polymers of different natures, accompanied by gelation [4,5,6]. As a result of this process, spatial structures with the characteristics of solids are formed. An important aspect of gelation is the ability to control the structure of the resulting material. During the polymer combination, intermacromolecular interactions dominate, which contributes to the realization of self-organizational capabilities and a significant improvement in the properties of the initial polymers. Such targeted modification opens up broad prospects for the use of composite materials in various fields of science and technology, including catalysis [7,8], medicine [9,10,11,12], bioengineering, the textile and paper industry [13], and the processes of extraction, separation, and concentration of metal ions and organic substances [14,15,16,17,18].

Poly(vinyl alcohol) (PVA), which has a high ability to form structures, is one of the most widely studied polyelectrolytes in the creation of composites. This feature is actively used in various industries. Poly(vinyl alcohol) is of great interest as a gelling agent due to the presence of a significant number of hydroxyl groups [19,20,21]. According to the literature, dilute solutions of PVA are true thermodynamically stable solutions, whereas concentrated solutions are unstable [22,23]. When heated, PVA undergoes complex chemical transformations, including intra- and intermolecular dehydration reactions. Intramolecular dehydration leads to the formation of cyclic units in macromolecules, whereas intermolecular dehydration forms units constructed like simple vinyl esters, which gives the polymer a mesh structure.

One of the directions of formation of the branched crosslinked structures of PVA occurs due to the formed intermolecular bonds of a physical nature. However, the self-association of alcohol macromolecules is difficult. The structural reorganization of polymers in aqueous solutions at the molecular and supramolecular levels can be carried out by changing the properties of the medium by modifying their polymer chains, which, in turn, will lead to a change in the rheological characteristics of polymers. The structure formation of aqueous solutions of PVA can be controlled using various modifying additives, in particular, sodium humate.

Previously, we obtained new polymer composites based on humic acids using natural polymer (gelatin) and synthetic polymer (poly(vinyl alcohol), urea-formaldehyde resin) for the purification of mine waters in the Karaganda region [24]. It was found that, when combining solutions of the components, various intermolecular interactions arise that contribute to the implementation of the self-organizing properties of humic acids and the polymers interacting with them. Tests have shown that the degree of cation removal from wastewater using the obtained composites ranges from 54 to 80% depending on their composition. The composites are comparable in efficiency with synthetic ion exchangers, while they are distinguished by the availability of raw materials and the simplicity of the production technology, which makes them promising for use as sorbents. Despite a sufficient number of studies on the properties of PVA, as well as the presence of works devoted to its gelation in the presence of various substances, the issues of the emergence of spatial structures of a mixture of poly(vinyl alcohol) with sodium humate have not been studied. Given the high chemical potential of humates [24,25], as well as the lack of systematic research in the field of obtaining and studying the characteristics of composites based on poly(vinyl alcohol) and sodium humate, it seems relevant to conduct research in this direction.

One of the ways to improve the quality of composite materials is by modifying polymers in a common solvent, which is widely used in various industries. The physico-chemical properties of such composites differ significantly from additive composites and are stable during operation. The production of composites by mixing polymers is a promising modification method and an effective way to improve their properties. The formation of an inner mesh as a result of polymer complexation enhances the valuable properties of the starting materials and gives them new ones. The viscometric method is used to evaluate complexation in mixtures of polymer solutions, since it is the simplest in experimental terms. The study of changes in relative viscosity over time makes it possible to identify the presence of structuring phenomena in dilute mixed systems.

In this regard, the aim of the work is to study the effect of sodium humate on the structure formation of poly(vinyl alcohol) and to study its physico-chemical characteristics.

## 2. Materials and Methods

### 2.1. Materials

In the synthesis of composite materials, we used the following: 5% aqueous solution of humic acid salts, sodium humate (HNa), isolated from oxidized coal from the Shubarkol deposit (Karaganda, Kazakhstan); 5% aqueous solution of poly(vinyl alcohol) (PVA, M_w_ 89,000–98,000, 99+% hydrolyzed. Merck, Darmstadt, Germany; CAS Number: 9002-89-5); 4% aqueous solution of sodium hydroxide (NaOH) (Merck, Darmstadt, Germany; CAS:1310-73-2); and 15% aqueous solution of hydrochloric acid (HCl). (Ridder, Karaganda, Kazakhstan).

### 2.2. Synthesis of Sodium Humate

Sodium humate (HNa) is obtained from humic acids isolated from oxidized coal from the Shubarkol deposit by the following method: 25 mL of water is added to 10 g of oxidized coal with stirring for good wetting, then 25 mL of a 4% NaOH solution. The extraction process is continued for two hours at the boiling point of a water bath. Next, the mixture is diluted with 20 mL of distilled water and we continued extraction at the same temperature for half an hour. The solution is allowed to settle for 3 h, and then the filler solution is carefully drained. The residual coal is treated with 20 mL of water and the mixture is heated at a turning and boiling point of a water bath for 30 min. The first and second filtrates of sodium humate solutions are combined. The resulting aqueous solution of sodium humate is acidified with a 15% HCl solution to pH 3. After settling, the precipitated humic acid is centrifuged, washed on a filter with distilled water to a negative sample of washing water with silver nitrate, and dried to a constant weight in a drying cabinet at 60 °C. The yield of humic acids is 70%, depending on the batch of oxidized coal. Next, 10 mL of 0.1 N NaOH is added to 1 g of humic acid with stirring. Mixing is carried out on a magnetic stirrer until the humic acid is completely dissolved. After the humic acid is completely dissolved, the sodium humate (HNa) solution is poured into a Petri dish and dried at a temperature of 60 °C.

### 2.3. Synthesis of Composite Material

To synthesize the composite material, the method of mixing 5% aqueous solutions of PVA and HNa was used, the content of which varied from 0 to 10 (%, wt.) in relation to poly(vinyl alcohol). The components were mixed on a magnetic stirrer for 120 min. Next, the reaction mixture was left in air at 25 °C to evaporate the solvent. Light-brown and dark-brown films were obtained.

### 2.4. Methods for Studying the Hydrodynamic Properties of a Composite

To determine the kinematic viscosity of the obtained composites, a capillary glass viscometer of the VPZ-2 type with a capillary diameter of 0.56 mm was used. It is a U–shaped tube with a capillary soldered into its elbow. The measurement of viscosity using a capillary viscometer is based on determining the expiration time of a certain volume of liquid from a measuring tank through a capillary. To measure the time of the liquid flow, a rubber tube is placed on the discharge tube. Next, holding the knee with your finger and turning the viscometer over, lower the knee into a vessel with liquid and suck it with a rubber bulb to the mark M_2_, making sure that no air bubbles form in the liquid. At the moment when the liquid level reaches the mark of M_2_, the viscometer is removed from the vessel and quickly turned over to its normal position. Excess fluid is removed from the outside of the ends of the knee and a rubber tube is placed on it. The viscometer is installed in the thermostat so that the expansion is below the liquid level in the thermostat. After holding in the thermostat for at least 15 min at a set temperature, the liquid is sucked into the knee to about one third of the expansion height. Connect the knee to the atmosphere and determine the time of movement of the meniscus fluid from M_1_ to M_2_. The relative viscosity is calculated by the following formula:

(1) $$\eta_{Rel}=\frac{\tau_{Sol-n}}{\tau_{Sol-t}}$$

where *η_Rel_*—the value of relative viscosity; *τ_Sol-n_*—the expiration time of a certain volume of solution (s); and *τ_Sol-t_*—the expiration time of a certain volume of solvent (s).

### 2.5. Determining the Structuring Time

To determine the structuring time, the sample retention time, which characterizes the loss of fluidity at which the liquid stops flowing, was taken as the temporary structuring criterion. To measure it, the sample is placed in a glass in such a way that the layer height does not exceed 5 cm, cooled at a set speed to 20 °C, and placed in a thermostat. The system is analyzed at certain time intervals. If the test material is held at an angle of 90 °C for 5 s without movement, then this time is fixed as the structuring time.

### 2.6. Adsorption of Cu^2+^ Ions

The sorption of the composite was carried out in a static mode. To do this, a weight of 1 g was placed in a flat-bottomed flask with a ground stopper. Next, 100 mL of copper sulfate solutions with an increasing concentration of 10–150 mmol/L was poured into the flasks. The solutions were mixed for 24 h using a laboratory shaker (PE-6410, Saint Petersburg, Russia). After the adsorption equilibrium was established, the product was separated by filtration. The concentrations of Cu^2+^ ions in the filtrate were determined using an iCAP6500 inductively coupled plasma atomic emission spectrometer (SPECTRO ARCOS EOP SPECTRO Analytical instruments GmbH, Kleve, Germany).

The amount of Cu^2+^ ions adsorbed on the surface of the composite was calculated as the ratio of the difference in concentrations of Cu^2+^ ions in the solution before and after sorption multiplied by the volume of the solution related to the unit mass of the composite:

(2) $$A=\frac{\left(C_{0}-C_{eq}\right)\cdot V}{m}$$

where A—the number of sorbed Cu^2+^ ions (mmol/g); *C*_0_—the concentration of Cu^2+^ ions in the initial solution before sorption (mmol/L); *C_eq_*—the equilibrium concentration of Cu^2+^ ions in the solution after sorption (mmol/L); m—the mass sorbent (g); and *V*—the volume of the analyzed solution (L).

The composition of HNa and synthesized composites is confirmed by IR spectroscopy data performed on the FSM-1201 IR Fourier spectrometer (Infraspec Company, St. Petersburg, Russia) in the range of wave numbers 4000–400 cm^−1^.

The surface topography of HNa and composites was studied using a high-resolution atomic force microscope (AFM) JSPM-5400 (JEOL, Tokyo, Japan). The morphology of the surface and the roughness of the samples were analyzed from AFM images.

The equilibrium concentrations of Cu^2+^ ions in the filtrate were determined using an inductively coupled plasma iCAP6500 atomic emission spectrometer (SPECTRO ARCOS EOP SPECTRO Analytical instruments GmbH, Kleve, Germany).

## 3. Results and Discussion

The synthesis of composites was carried out by mixing aqueous solutions of poly(vinyl alcohol) and sodium humate, the content of which varied from 2 to 10 (wt. %) in relation to poly(vinyl alcohol). The components were mixed on a magnetic stirrer for 120 min.

In the course of preliminary experiments, it was found that sodium humate affects the critical stability of jelly formation in aqueous solutions of poly(vinyl alcohol). During the research, the initial concentration of the mixed solutions was determined, equal to 5% with the introduction of sodium humate in an amount of 10% of the PVA.

**Figure 1:**
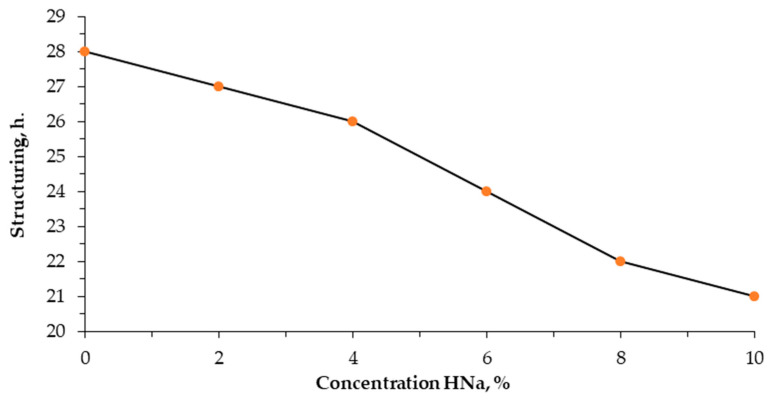
Effect of sodium humate content on structuring time 5% aqueous solution of poly(vinyl alcohol) at 20 °C.

An analysis of the structuring time showed that sodium humate has a certain effect on the formation of the structure of poly(vinyl alcohol) (Figure 1).

It follows from Figure 1 that, in the absence of sodium humate, the structuring process of aqueous PVA solutions takes 28 h. With an increase in the sodium humate content from 2 to 6%, the structuring time decreases to 24 h. Its most pronounced effect is observed with the introduction of 8–10% sodium humate, while the structuring time is reduced to 21 h.

Studies have shown that the formed systems are characterized by a homogeneous structure, without signs of stratification. Sodium humate, being a polymer compound with ionogenic groups, is capable of acting as a modifier of poly(vinyl alcohol) structure formation. Its effect is probably due to a combination of several factors: electrostatic interaction, changes in hydrogen bonds, and the coagulation effect.

The results of the study indicate that reducing the structuring time of poly(vinyl alcohol) with the introduction of sodium humate to 10% plays a key role in technological processes where the accelerated formation of gel structures and control of the properties of PVA gels are necessary. It also helps to improve the performance characteristics of materials based on it.

**Figure 2:**
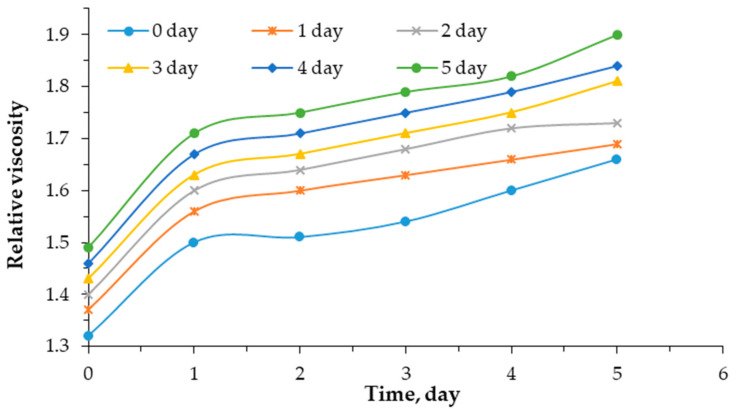
Kinetics of changes in the relative viscosity of 0.5% mixed aqueous solution of poly(vinyl alcohol)—sodium humate as a function of time at 20 °C.

**Figure 3:**
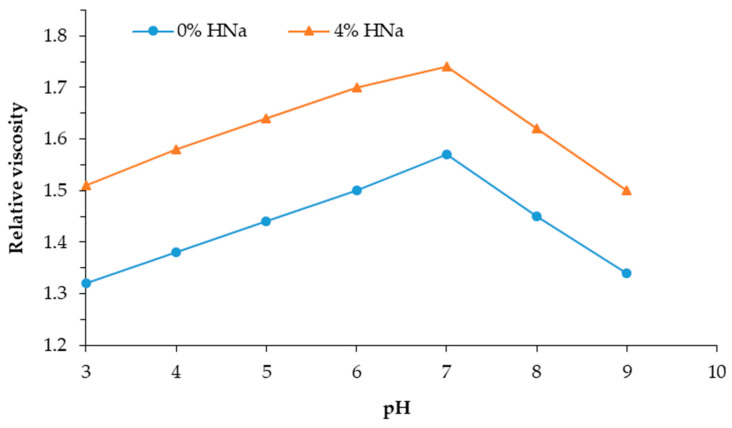
Dependence of the relative viscosity of aqueous mixtures of poly(vinyl alcohol)—sodium humate on the pH of the mixture at 20 °C.

Figure 2 and Figure 3 show the kinetics of changes in the relative viscosity of a 0.5% mixed aqueous solution of poly(vinyl alcohol) and sodium humate, depending on the sodium humate content and time.

The graph in Figure 2 illustrates the kinetics of changes in the relative viscosity of 0.5% aqueous PVA solution depending on time and sodium humate content. Regardless of the sodium humate content, the viscosity of the solution increases with time. Therefore, without sodium humate (0%), the initial viscosity is 1.32 mm^2^/s. The relative viscosity of the studied systems after a day ranged from 1.37 to 1.69 mm^2^/s. When the concentration of sodium humate increases to 10% after 3 days, the viscosity increases to 1.81 mm^2^/s. The greatest increase in viscosity is observed with the introduction of 10% sodium humate on day 5.

Thus, the higher the concentration of sodium humate over time, the viscosity of the PVA–HNa solution increases. Comparing the slope of the curves, it can be seen that, with a higher content of sodium humate, the slope becomes steeper. This indicates a more intense change in viscosity over time. On average, when 10% sodium humate is added, the viscosity of the mixture increases by 16.3%.

Based on the above, we can imagine the following principle of operation of the mechanism. Sodium humate is a mixture of high-molecular-weight compounds with active functional groups (carboxylic, phenolic, and hydroxyl) that can interact with PVA in various ways:

1. The formation of hydrogen bonds. Poly(vinyl alcohol) contains hydroxyl groups that can form hydrogen bonds with each other and with sodium humate. Carboxylic and phenolic groups of sodium humate enhance intermolecular bonds in solution, reduce molecular mobility, and increase viscosity.
2. Ionic interactions. Sodium humate, being an anionic polymer, can interact with cations in solution (for example, sodium ions), creating additional structuring points. This leads to an increase in molecular ordering and an increase in viscosity.
3. The solvation effect. Water molecules bind to both PVA and sodium humate, forming hydrate structures. As the sodium humate content increases, competition for water molecules increases, which leads to an increase in viscosity.
4. The aggregation and formation of a mesh structure. Over time, the molecular complexes become larger when the molecules of PVA and sodium humate begin to form a three-dimensional structure. This causes a further increase in viscosity, especially pronounced at high concentrations of sodium humate.

Figure 3 shows a graph of the dependence of the relative viscosity of a solution of PVA with sodium humate on the pH of the mixture.

The graphs in Figure 3 illustrate the kinetics of changes in the relative viscosity of a 0.5% aqueous PVA solution depending on the pH of the mixture. The structuring process in mixed PVA–HNa systems is kinetic in nature and slows down in the presence of HNa, which indicates the development of specific intermolecular interactions. The introduction of HNa into the PVA solution leads to an increase in the initial viscosity and a more intense growth of viscosity over time. It is noted that, with an increase in the concentration of HNa, the effect increases, which confirms its active participation in the formation of intermolecular networks.

The maximum increase in viscosity was recorded on the fifth day with the introduction of 10% sodium humate at pH = 7, which indicates optimal conditions for the formation of stable structures. The increase in viscosity over time indicates the gradual formation of dense molecular complexes caused by a complex of interactions: the hydrogen bonds, ionic interactions, and structural ordering of polymer chains.

In an acidic medium (pH < 5) of the solution, with an increase in the concentration of sodium humate, the viscosity increases. This is due to the fact that, in an acidic medium, the carboxyl groups of sodium humate are partially protonated, which reduces their ionization and electrostatic repulsion between macromolecules. As a result, the density of polymer chains increases, which leads to an increase in viscosity.

At alkaline pH > 8, the viscosity begins to decrease, since the carboxyl groups of sodium humate are completely ionized and electrostatic repulsion between the molecules occurs, which leads to the unfolding of the PVA chains and the destruction of structured bonds. As a result, the solution becomes less viscous.

The optimum viscosity is observed in a neutral media (pH = 7). The optimality of pH = 7 is explained by the fact that, under these conditions, a balance is achieved between the ionized and non-ionized forms of the functional groups of both PVA and HNa. At a neutral reaction of the environment, the carboxyl groups of HNa are predominantly in the ionized form, which facilitates electrostatic interaction with the hydroxyl groups of PVA. At the same time, the ability to form hydrogen bonds is preserved, since the pH is not too high to cause the complete deactivation of the donor–acceptor sites. The tendency toward the aggregation and phase separation of the components decreases.

Thus, the optimal pH range for the operation of the PVA:HNa composite is 7.0. A neutral environment promotes the maximum degree of interaction between the components, which is manifested in a significant increase in the viscosity and stabilization of the system structure.

**Figure 4:**
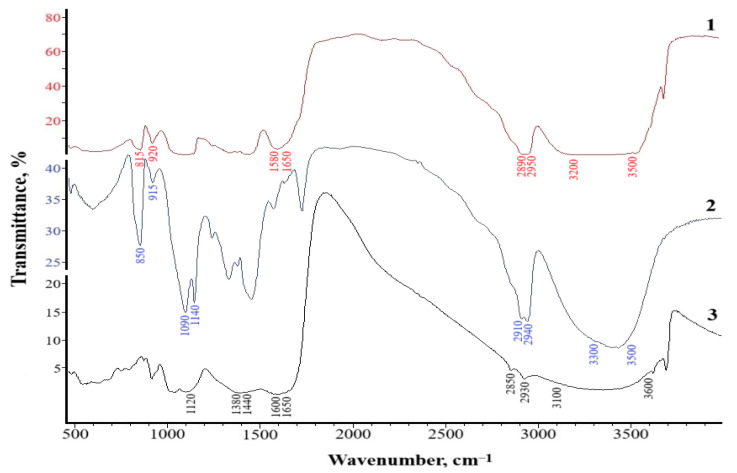
IR spectra: 1—composite PVA:HNa; 2—PVA; and 3—sodium humate.

Infrared (IR) spectroscopy was carried out to determine the nature of the intermolecular interactions in the composite based on PVA and HNa. Figure 4 shows the IR spectra of the original components and the resulting composite.

An analysis of the IR spectra of poly(vinyl alcohol) and sodium humate (Figure 4) indicates that they are characterized by typical wide absorption bands with a maximum at 3100–3600 cm^−1^, due to the presence of OH hydroxyl groups for both poly(vinyl alcohol) and sodium humate. Thus, poly(vinyl alcohol) is characterized by the presence of –OH groups in the range of 3300–3500 cm^−1^; for sodium humate, this group is observed in the range of 3100–3600 cm^−1^. Valence fluctuations in the C–H bond of aromatic and aliphatic groups for both PVA and HNa are manifested in the range of 2850–2950 cm^−1^. Asymmetric and symmetric valence vibrations of the C=O carboxyl bond are observed in the region of 1600–1650 cm^−1^ (asymmetry) and 1380–1440 cm^−1^ (symmetry) on the HNa spectra. Fluctuations in the 1080–1140 cm^−1^ range on the PVA spectra indicate the presence of ether groups –C–O–C and 1000–1300 cm^−1^ on the spectra of sodium humate, the presence of carbohydrates, and cyclic and aliphatic alcohols, as well as esters. The PVA spectra also contain absorption bands of valence vibrations of conjugated carbon double bonds C=C in the region of 850–900 cm^−1^ and pendulum vibrations of (CH_2_)_n_ fragments with *n* > 4 in the region of 600–900 cm^−1^ for sodium humate.

A comparative analysis of the spectra of the initial components and composites (Figure 4) revealed a number of characteristic changes indicating the formation of intermolecular interactions between the functional groups of the PVA and HNa components. In the region of 3200–3500 cm^−1^, responsible for the stretching vibrations of hydroxyl groups (–OH), the spectrum of the composite shows a shift of the band towards lower frequencies (3200 cm^−1^) and its significant broadening compared to the spectra of the initial components. These changes indicate the formation of hydrogen bonds between the –OH groups of PVA and the carboxyl (–COO^−^) or phenolic groups of HNa.

In the region of 1650 cm^−1^, characteristic of the stretching vibrations of C=O in the carboxyl and conjugated aromatic systems of HNa, a decrease in intensity and a slight shift of the band are noted in the spectrum of the composite, which may also indicate the involvement of carboxyl groups in electrostatic or hydrogen interactions. The characteristic bands of symmetric and asymmetric vibrations of the carboxylate anion (–COO^−^), located in the region of 1550 and 1400 cm^−1^, demonstrate a decrease in intensity and minor shifts in the positions of the maxima. This may indicate a change in the electron density on these groups due to the interaction with the hydroxyl groups of PVA and the formation of intermolecular complexes. Additionally, in the region of 1000–1150 cm^−1^, characteristic of C–O–C and C–O–H vibrations, changes in the intensity of the bands are observed, which also indicates the participation of the alcohol groups of PVA in interactions with the functional groups of HNa. The bands in the region of 600–900 cm^−1^ may indicate the interaction of the aromatic structures of sodium humate with the hydroxyl groups of PVA.

Thus, the analysis of the IR spectra allows us to conclude that hydrogen and electrostatic interactions between PVA and HNa are realized in the structure of the PVA–HNa composite. The main contribution to the formation of the structure is made by hydrogen bonds between –OH and –COO^−^ groups, which contributes to the formation of the spatial structure of the composite.

**Figure 5:**
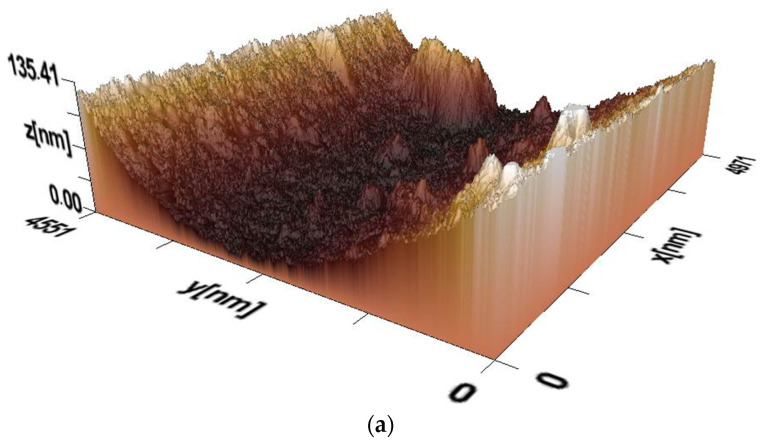

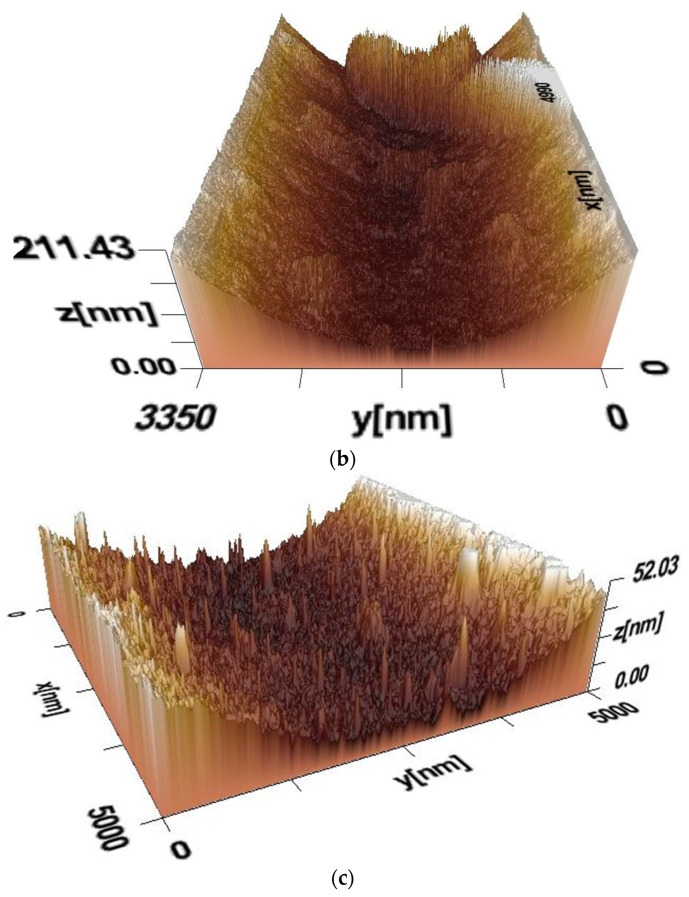
Three-dimensional image of the surface: (**a**)—PVA:HNa = 9:1; (**b**)—PVA:HNa = 8:2; and (**c**)—PVA:HNa = 7:3.

Figure 5a–c show 3D images of the surface morphology of films of composites PVA:HNa of different composition (mass.%): PVA:HNa = 9:1; PVA:HNa = 8:2; and PVA:HNa = 7:3, obtained at pH = 7. In this environment, sodium humate is in its most ionized state. Strong intermolecular bonds form between PVA and sodium humate and the surface becomes more pronounced and prominent.

**Figure 6:**
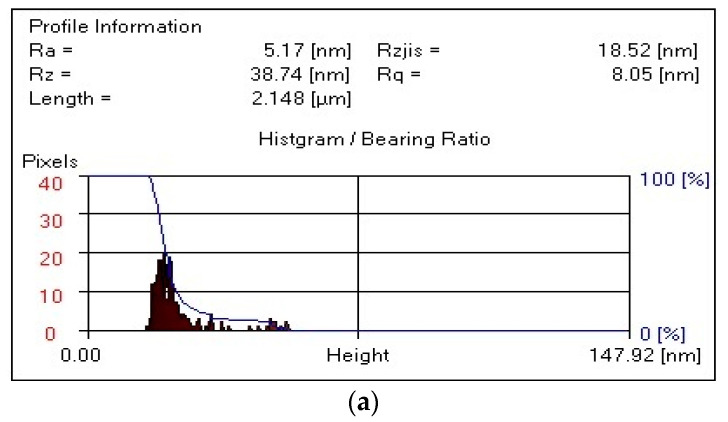

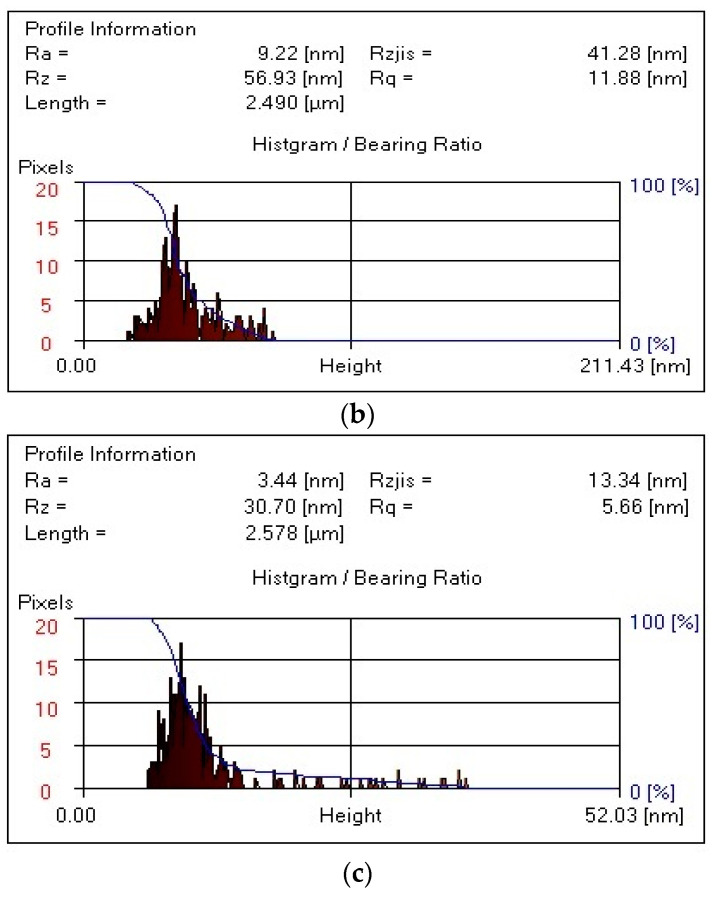
Roughness parameters: (**a**)—PVA:HNa = 9:1; (**b**)—PVA:HNa = 8:2; and (**c**)—PVA:HNa = 7:3.

Figure 5a shows that the surface of the composite film (PVA:HNa = 9:1) has pronounced irregularities, which indicates significant roughness. There is a deepening in the central region, which may be due to varying degrees of phase separation or the effect of the sodium humate concentration. The maximum relief height reaches approximately 135 nm. This indicates the presence of heterogeneous microstructures. Upon a closer examination of Figure 6a, smooth and rough zones are visually distinguished, which may be due to the different density of HNa distribution in the PVA. There are more pronounced ridge-like formations along the edges, which may be the result of crystallization. The presence of different morphological zones may indicate a partial incompatibility of HNa and PVA in this ratio. Deep areas may correspond to areas with a high concentration of HNa, which affects the moisture distribution in the material. The ridge-like structures at the edges may be the result of phase separation, when the PVA film concentrates in certain areas during drying. It follows from the above that the 3D image of the composite surface (PVA:HNa = 9:1) shows a non-uniform relief with varying roughness. The central depression and ridge-like edges may be related to the difference in hydrophilicity and phase separation of the components. The data obtained can be useful for the further analysis of component compatibility and the optimization of composite composition.

With an increase in the concentration of sodium humate in the composite (PVA:HNa = 8:2) compared with the previous composite (PVA:HNa = 9:1), the maximum height of the relief has increased and reaches approximately 211 nm, indicating an increase in roughness. As in the previous case, in the composite (PVA:HNa = 8:2), there is a deep depression, which may indicate a heterogeneous distribution of the components. A more pronounced depth may indicate the aggregation of HNa, which is unevenly distributed in the PVA matrix. There are also sharp protrusions along the edges of the 3D image, which may indicate the formation of hard areas, probably associated with the crystallization or accumulation of PVA. The overall increase in relief and pronounced protrusions may be the result of increased phase separation. Thus, an increase in roughness with an increase in the concentration of PVA indicates a decrease in the compatibility of the components. The central recess may indicate areas with a high concentration of PVA, which affects the moisture and film structure. Ridge-like protrusions at the edges may be associated with the drying process, in which PVA concentrates in certain areas.

In comparison with previous samples, the surface of the film of the composite PVA:HNa = 7:3 becomes more uniform, but still retains a pronounced roughness. The maximum relief height reaches approximately 52 nm, which is significantly less than in the samples PVA:HNa = 9:1 and PVA:HNa = 8:1. This may indicate a more uniform distribution of PVA in the polymer matrix at a neutral pH. Unlike the previous images, the deep indentations and sharp protrusions are less pronounced. This may indicate the improved compatibility of HNa and PVA at pH = 7. Moreover, instead of large aggregations, a fine-grained surface structure is observed, which may be due to the uniform distribution of PVA in the polymer matrix. A high density of small protrusions may indicate the presence of hydrogen bonds and intermolecular interactions that stabilize the surface. A decrease in the surface at pH = 7 may also be associated with a more uniform spreading of the PVA and a decrease in phase separation. The absence of deep and sharp peaks indicates the increased compatibility of the components in this pH. The fine granular structure may indicate the stability of the film due to the optimal interaction of the functional groups of HNa and PVA.

Figure 6a–c represent the roughness parameters of the obtained composites of different compositions (wt. %): PVA:HNa = 9:1; PVA:HNa = 8:2; and PVA:HNa = 7:3, obtained at pH = 7. The roughness parameters shown in Figure 6 include the following: the arithmetic mean roughness (R_a_), maximum roughness height (R_z_), and RMS roughness (R_q_). R_a_ characterizes the average amount of deviations in the surface height from its ideal flat line. It measures how “uneven” the surface is overall. R_z_ measures the difference between the highest and lowest point on the surface, which allows you to understand the maximum depth and height of the irregularities. R_q_ is the standard deviation of the surface height and serves as a more accurate measure of roughness than R_a_, especially in the case of complex surface profiles.

It follows from Figure 6 that, for the composite PVA:HNa = 9:1 (Figure 6a), the R_a_ value is significantly reduced to 5.17 nm, which indicates a smoother surface compared to the initial HNa. For the composite PVA:HNa = 8:2 (Figure 6b), the R_a_ value increases to 9.22 nm, which indicates a slight deterioration in the smoothness of the surface compared to the composite PVA:HNa = 9:1. For the composite PVA:HNa = 7:3 (Figure 6c), this value is 3.44 nm, which is the smallest among all variants, indicating the smoothest surface among the samples considered.

The maximum roughness height of the composite PVA:HNa = 9:1 is significantly reduced to 38.74 nm, which indicates a smoother surface. For the composite PVA:HNa = 8:2, this value is 56.93 nm, which is slightly higher than that of PVA:HNa = 9:1, indicating a more pronounced roughness. The composite PVA:HNa = 7:3 has an R_z_ value of 30.70 nm, which is the minimum value among composites PVA:HNa, indicating the smoothest surface in this row.

The RMS roughness value (R_q_) for the composite PVA:HNa = 9:1 is significantly lower at 8.05 nm, which also confirms the improvement in surface smoothness. For composite PVA:HNa = 8:2, the value of R_q_ is 11.88 nm, which is slightly higher than that of PVA:HNa = 9:1. The R_q_ value of the composite PVA:HNa = 7:3 is equal to 5.66 nm, which is again the lowest among all composites, confirming that this sample has the greatest smoothness.

Thus, the composite PVA:HNa = 9:1 composition has the highest values of all roughness parameters. An increase in the sodium humate content in the case of PVA:HNa = 8:2 leads to an increase in roughness, but it still remains less rough. The composite PVA:HNa = 7:3 has the lowest values of all parameters, which indicates the greatest smoothness among all the studied composites. Increasing the HNa content in the system significantly improves the surface characteristics, reducing its roughness.

**Figure 7:**
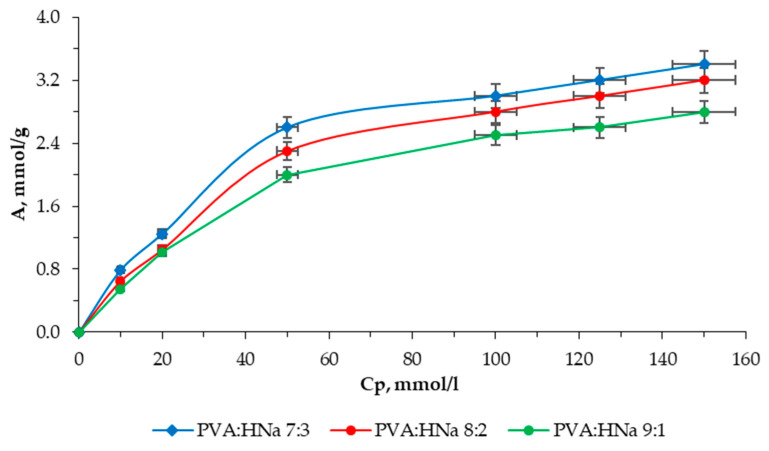
Isotherms of Cu^2+^ ion sorption using composites.

Figure 7 shows the sorption isotherm for a composite (PVA:HNa = 7:3).

Based on experimental data, it has been established that the composite of the composition of PVA:HNa = 7:3 has a higher sorption capacity compared to composites of compositions PVA:HNa = 9:1 and PVA:HNa = 8:2. The limiting sorption for the composite PVA:HNa = 7:3 was 3.40 mmol/g.

**Figure 8:**
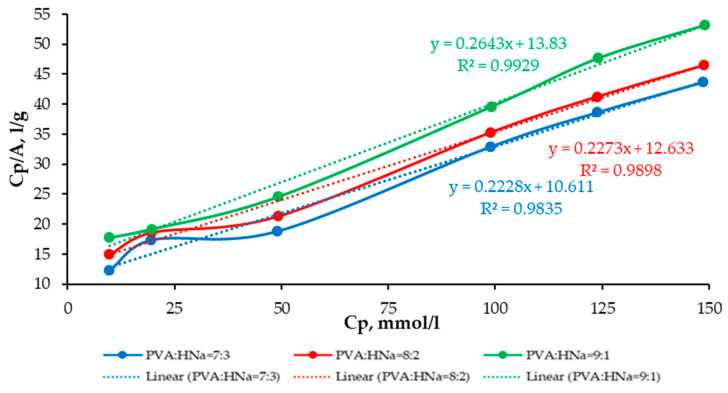
The isotherm of the sorption of Cu^2+^ ions on composites PVA:HNa in the coordinates of the linear form of the Langmuir equation.

**Figure 9:**
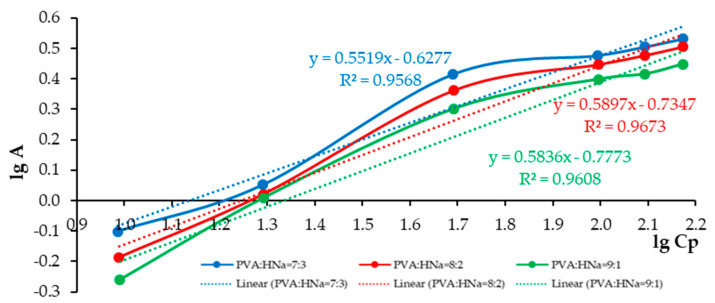
The isotherm of the sorption of Cu^2+^ ions on composites PVA:HNa in the coordinates of the linear form of the Freundlich equation.

A comparative analysis of the sorption characteristics of three different polymer composite compositions is presented as follows: PVA:HNa = 9:1; PVA:HNa = 8:2; and PVA:HNa = 7:3. The assessment is carried out using the Langmuir and Freundlich isotherms. The results are presented in Figure 8 and Figure 9 and Table 1 and Table 2.

The analysis of the experimental data approximated by the Langmuir model demonstrates that, with an increase in the proportion of HNa in the composite, a decrease in the parameters k and b is observed, which indicates a denser saturation of the surface with active centers and an increase in the affinity for the sorbate. The polymer composite PVA:HNa = 7:3 shows a higher limiting adsorption (A_∞_)—4.4884 mmol/g compared to other compositions and a significantly higher K_L_—0.021 L/mmol. The analysis of experimental data approximated by the Freundlich model showed that changing the ratio of polymer composites also significantly affects the sorption characteristics. The best fit of the model (the highest value of the determination coefficient R^2^ = 0.9673) was achieved at a ratio of 8:2, which indicates the most adequate description of the sorption process by the Freundlich model for this composition. The maximum value of the sorption intensity index n = 1.8120 was recorded at a ratio of 7:3, indicating a high degree of heterogeneity of the sorption surface and sorption efficiency at low concentrations. The highest sorption capacity K_F_ = 2.8424 was observed at a ratio of 9:1. Thus, the choice of the optimal composition of the polymer composite should be based on priorities: for accurate modeling—8:2, for high sorption intensity—7:3, and for maximum sorption capacity—9:1.

## 4. Conclusions

This study presents the synthesis and characterization of composites by mixing based on aqueous solutions of poly(vinyl alcohol) and sodium humate used as an absorption material for removing Cu(II) metal ions from water. The concentrations of sodium humate in the composite ranged from 2 to 10 (wt. %) in relation to poly(vinyl alcohol). The patterns and features of the structure formation process in mixed aqueous solutions of poly(vinyl alcohol) with sodium humate have been studied. It is shown that the structuring of mixed systems is a kinetic process that slows down in the presence of sodium humate. Sodium humate increases the initial viscosity of the PVA solution and accelerates its growth over time. The higher the concentration of sodium humate, the more pronounced the effect observed. The greatest increase in viscosity is observed with the introduction of 10% sodium humate at pH = 7 on day 5. The main mechanisms of interaction are hydrogen bonds, ionic interactions, solvation, and structural ordering. An increase in viscosity indicates the gradual formation of dense molecular complexes in solution. The morphology of the surface of the obtained composites has been studied. The 3D image of the surface PVA:HNa = 7:3 shows a significantly uniform morphology compared to the samples PVA:HNa = 9:1 and PVA:HNa = 8:1. This confirms that, at pH = 7, it contributes to improved component compatibility, reducing phase separation and the formation of large aggregates. Such a composite probably has better mechanical properties, which can be useful in application. In tasks related to water purification and Cu(II) removal, the composite of the composition PVA:HNa = 7:3 demonstrates good performance due to the active centers formed during the structure formation process. The maximum adsorption of Cu^2+^ ions for the composite was 3.40 mmol/g. The resulting material can be used to remove Cu(II) from water resources.

## Figures and Tables

**Table 1:** Linear approximation coefficients (y = kx + b), Langmuir equation parameters, and correlation coefficients (r).

Composite	Ratio	k	b	Maximum Specific Adsorption, A_∞_, mmol/g	Adsorption Equilibrium Constant, K_L_, L/mmol	r
PVA:HNa	7:3	0.2228	10.6108	4.4884	0.0210	0.9835
	8:2	0.2273	12.6329	4.3999	0.0180	0.9898
	9:1	0.2643	13.8303	3.7832	0.0191	0.9929

**Table 2:** Linear approximation coefficients (y = kx + b), Freundlich equation parameters and correlation coefficients (r).

Composite	Ratio	k	b	K_Fr_	n	r
PVA:HNa	7:3	0.5519	−0.6277	2.2203	1.8120	0.9568
	8:2	0.5897	−0.7347	2.7120	1.6957	0.9673
	9:1	0.5836	−0.7773	2.8424	1.7134	0.9608

## Data Availability

The original contributions presented in this study are included in the article. Further inquiries can be directed to the corresponding author.
